# Scope withdrawal technique to prevent transesophageal puncture during endoscopic ultrasound-guided hepaticogastrostomy

**DOI:** 10.1055/a-2623-6215

**Published:** 2025-07-02

**Authors:** Kazuki Endo, Haruo Miwa, Ritsuko Oishi, Hiromi Tsuchiya, Yuichi Suzuki, Kazushi Numata, Shin Maeda

**Affiliations:** 1 26437 Gastroenterological Center, Yokohama City University Medical Center, Yokohama, Japan; 2 Department of Gastroenterology, Yokohama City University Graduate School of Medicine, Yokohama, Japan

In endoscopic ultrasound-guided hepaticogastrostomy (EUS-HGS), puncture of the intrahepatic bile duct segment B2 is considered to make guidewire and device insertion easier than B3 puncture. However, B2 puncture carries a risk of esophageal puncture, which can lead to severe complications such as mediastinitis 1 2 . To avoid esophageal puncture, several techniques have been reported 3 4 5 ; however, there are no reports focusing on puncture techniques. Herein, we report a novel puncture technique, termed the “scope withdrawal technique,” to prevent transesophageal puncture during EUS-HGS ( Video 1 ).

Scope withdrawal technique to prevent transesophageal puncture during B2 puncture during endoscopic ultrasound-guided hepaticogastrostomy.Video 1

A 70-year-old man with pancreatic carcinoma was admitted with cholangitis caused by occlusion of a self-expandable metal stent (SEMS) in the common bile duct. Endoscopic retrograde cholangiopancreatography was performed; however, additional SEMS placement failed due to tumor invasion into the duodenum. Endoscopic nasobiliary drainage was initially performed, and EUS-HGS was carried out after the cholangitis improved.

When B2 puncture is attempted, the portal vein may obstruct the puncture route. Withdrawing the scope to avoid the portal vein and target a more peripheral bile duct increases the risk of esophageal puncture. To address this issue, we first punctured the gastric wall slightly distal to the intended puncture line using a 19G fine-needle aspiration needle (EZ Shot 3 Plus; Olympus, Tokyo, Japan). Keeping the needle in place, the scope was withdrawn to adjust the puncture line without changing the needle entry point in the gastric wall ( Fig. 1 ). After B2 puncture and guidewire insertion, a fully covered SEMS (Niti-S EUS-BD system, End Bare Single Flare, 8 mm × 10 cm; Taewoong Medical Co., Ltd., Gimpo, South Korea) was placed. The SEMS was placed just below the esophagogastric junction, and a CT scan on the day after the procedure confirmed transgastric placement ( Fig. 2 ).

**Fig. 1:**
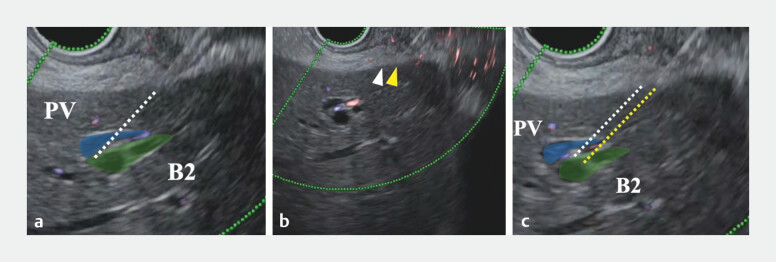
Scope withdrawal technique to prevent transesophageal puncture during intrahepatic bile duct segment B2 puncture. **a** The gastric wall is punctured slightly distal to the intended site, penetrating only the gastric wall and stopping just before reaching the liver surface (white dotted line: puncture line). **b** The scope is withdrawn while keeping the needle in place (the tip of needle moves from the white arrowhead to the yellow arrowhead). **c** The puncture line is adjusted from the white to the yellow dotted line without changing the gastric entry point.

**Fig. 2:**
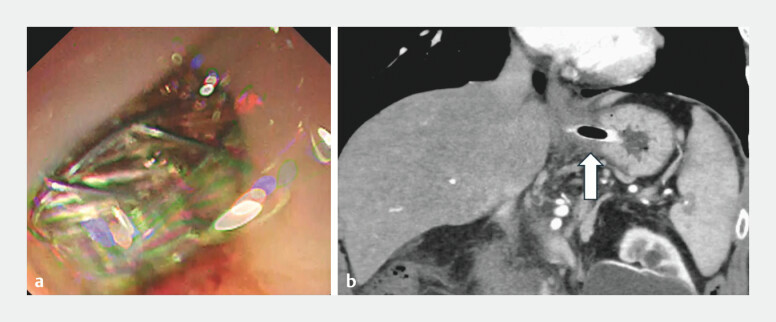
The position of the self-expanding metal stent (SEMS) after endoscopic ultrasound-guided hepaticogastrostomy. **a** The SEMS is placed just below the esophagogastric junction. **b** A CT scan on the day after the procedure reveals that the SEMS is deployed from the stomach.

To the best of our knowledge, this is the first report of a puncture technique to prevent esophageal puncture during EUS-HGS.

Endoscopy_UCTN_Code_TTT_1AS_2AH

## Notes

Endoscopy E-Videos https://eref.thieme.de/e-videos
*E-Videos* is an open access online section of the journal *Endoscopy* , reporting on interesting cases and new techniques in gastroenterological endoscopy. All papers include a high-quality video and are published with a Creative Commons CC-BY license. Endoscopy E-Videos qualify for HINARI discounts and waivers and eligibility is automatically checked during the submission process. We grant 100% waivers to articles whose corresponding authors are based in Group A countries and 50% waivers to those who are based in Group B countries as classified by Research4Life (see: https://www.research4life.org/access/eligibility/ ). This section has its own submission website at https://mc.manuscriptcentral.com/e-videos .
